# Supplementary material for: Epigenome-wide meta-analysis identifies DNA methylation biomarkers associated with diabetic kidney disease
Source: Nat Commun. 2022 Dec 22;13:7891. doi: 10.1038/s41467-022-34963-6 (PMC9780337; doi:10.1038/s41467-022-34963-6)

Epigenome-wide meta-analysis identifies DNA methylation biomarkers associated with diabetic kidney disease

Laura J Smyth[1]*, Emma H Dahlström[2,3,4]*, Anna Syreeni[2,3,4], Katie Kerr[1], Jill Kilner[1], Ross Doyle[5], Eoin Brennan[5], Viji Nair[6], Damian Fermin[7], Robert G Nelson[8], Helen C Looker[8], Christopher Wooster[1], Darrell Andrews[5], Kerry Anderson[1], Gareth J McKay[1], Joanne B Cole[9,10,11], Rany M Salem[12], Peter J Conlon[13], Matthias Kretzler[14], Joel N Hirschhorn[9,10,15], Denise Sadlier[16], Catherine Godson[5], Jose C Florez[9,11,17], Carol Forsblom[2,3,4], Alexander P Maxwell[1,18], Per-Henrik Groop[2,3,4,19], Niina Sandholm[2,3,4], Amy Jayne McKnight[1] on behalf of the GENIE consortium

*These authors contributed equally

Supplementary Tables

Supplementary Table 1 Summary of the population cohort characteristics and missing data per variable.....................2
Supplementary Table 2 Details of samples and CpGs retained in each analysis model during RnBeads QC................3
Supplementary Table 3 Look-ups of DKD-associated CpGs (p<9.9x10^{-8}) in EWAS on DKD in the CRIC study 4
Supplementary Table 4 eFORGE-TF results (p<x10^{-5}) for motif overlap with DKD-associated differentially methylated CpG sites..........5
Supplementary Table 5 Differential kidney gene expression for DKD in the NephroSeq database v4...........................6
Supplementary Table 6 Differential kidney gene expression in the North Dublin Renal Biobank RNA-Seq data............7
Supplementary Table 7 Kidney gene expression correlations in American Indian T2D population with DKD (n=97) 8
Supplementary Table 8 Mendelian randomisation results for the association between methylation at DKD-associated CpGs and DKD 9
Supplementary Table 9 Differentially methylated genes derived from METAL analysis from three models 9
Supplementary Table 10 Genetics of Nephropathy – an International Effort (GENIE) members 10

Supplementary Figures

Supplementary Figure 1 Representative concordance plots for a duplicate sample pair...13
Supplementary Figure 2 Flow chart depicting the methods undertaken..13
Supplementary Figure 3 Regional association plots (A-G) for the seven CpGs EWAS-significant in each meta-analysis model..........13
Supplementary Figure 4 Regional association plots for the DKD-associated CpG located within the *REV1* gene 18

Supplementary Tables

Supplementary Table 1 Summary of the population cohort characteristics and missing data per variable

Demographics	UK-ROI (*n*=504)	*n* (%)	FinnDiane (*n*=800)	*n* (%)
Sex, Male (%)	264 (52.4)	504 (100)	300 (37.5)	800 (100)
Age, years (SD)	41.6 (9.52)	504 (100)	43.4 (10.9)	800 (100)
Diabetes onset age, years (SD)	15.2 (7.3)	458 (90.9)	13.7 (8.4)	800 (100)
Diabetes duration, years (SD)	26.8 (7.38)	458 (90.9)	29.7 (9.2)	800 (100)
CD8+ T-cells, % (SD)	4.1 (4.3)	504 (100)	5.2 (4.4)	800 (100)
CD4+ T-cells, % (SD)	13.4 (6.4)	504 (100)	10.6 (4.9)	800 (100)
Natural killer cells, %	3.6 (4.5)	504 (100)	3.7 (4.6)	800 (100)
B-cells, %	3.2 (2.5)	504 (100)	3.5 (2.7)	800 (100)
Monocytes, %	8.2 (3.7)	504 (100)	7.7 (2.9)	800 (100)
Granulocytes, %	64.6 (11.4)	504 (100)	65.3 (9.1)	800 (100)
Smoking status, current (%)	89 (23.6)	377 (74.8)	203 (25.4)	800 (100)
HbA_{1c}, percentage (SD)	8.7 (1.9)	431 (85.5)	8.5 (1.5)	789 (98.6)
BMI, kg/m^2 (SD)	27.8 (7.3)	290 (57.5)	26.0 (4.0)	797 (99.6)
HDL cholesterol, mmol/mol (SD)	1.6 (0.5)	204 (40.5)	1.3 (0.4)	794 (99.3)
Triglycerides, mmol/mol (SD)	1.6 (1.5)	249 (49.4)	1.4 (1.0)	799 (99.9)

Continuous variables are reported as mean (standard deviation), and categorical variables are reported as number (%). Abbreviations: BMI=body mass index, FinnDiane=Finnish Diabetic Nephropathy study, HbA_{1c}=glycated haemoglobin, HDL=high-density lipoprotein, *n*=number, ROI=Republic of Ireland, SD=standard deviation, UK=United Kingdom

Supplementary Table 2 Details of samples and CpGs retained in each analysis model during RnBeads QC

Model	Variables adjusted for	Cohort	Pre RnBeads-QC and preprocessing stages: Initial sample (*n* included) *	Pre RnBeads-QC and preprocessing stages: Initial CpGs (*n* included)	Post RnBeads-QC and preprocessing stages: Final sample (*n* retained)	Post RnBeads-QC and preprocessing stages: Final CpGs (*n* retained)
Minimal model (*n*=1,302 after QC)	Age, sex and six WCCs	UK-ROI	504	866 895	497	764 643
		FinnDiane	798	866 895	797	763 064
Minimal Model plus current smoking status (*n*=1,175 after QC)	Age, sex, six WCCs and current smoking status	UK-ROI	377	866 895	372	768 287
		FinnDiane	798	866 895	797	763 064
Maximal model (*n*=957 after QC)	Age, sex, six WCCs, current smoking status, HbA_{1c}, HDL, triglycerides, duration of diabetes and BMI	UK-ROI	159	866 895	156	776 504
		FinnDiane	798	866 895	797	763 064

*after initial sex checks and removal of individuals with missing covariates. Abbreviations: BMI=body mass index, FinnDiane=Finnish Diabetic Nephropathy study, HbA_{1c} =glycated haemoglobin, HDL=high-density lipoprotein, *n*=number, QC=Quality control, ROI=Republic of Ireland, UK=United Kingdom

Supplementary Table 3 Look-ups of DKD-associated CpGs ($p<9.9x10^{-8}$) in EWAS on DKD in the CRIC study

CpG	Gene	HbA_{1c} *p*	p_{adj}	Albuminuria *p*	p_{adj}	eGFR *p*	p_{adj}	eGFR slope *p*	p_{adj}
cg10072464	*ADPRHL1*	0.075	1	0.193	1	0.002	0.256	0.216	1
cg22815707	*ANKRD12*	0.950	1	0.295	1	0.401	1	0.59	1
cg12378834	*C5orf66*	0.376	1	0.883	1	0.99	1	0.852	1
cg05325763	*CPT1A*	0.959	1	0.112	1	0.747	1	0.291	1
cg17058475	*CPT1A*	0.315	1	0.009	1	0.702	1	0.366	1
cg03546163	*FKBP5*	3.00E-03	0.384	0.891	1	0.151	1	0.21	1
cg05284887	*GJA5*	0.668	1	0.77	1	0.355	1	0.603	1
cg13125822	*GRK5*	0.274	1	0.532	1	0.737	1	0.97	1
cg18376497	*INPP4B*	0.604	1	0.493	1	0.005	0.64	0.387	1
cg02841972	*GRHL1, KLF11*	0.849	1	0.105	1	0.663	1	0.264	1
cg19996939	*HBS1L, MYB*	0.141	1	0.446	1	0.174	1	0.931	1
cg01895164	*PAFAH2, EXTL1*	0.654	1	0.863	1	0.308	1	0.837	1
cg12230203	*PTGIS, B4GALT5*	NA	NA	NA	NA	NA	NA	NA	NA
cg25544931	*ZNF763, ZNF433-AS1*	0.698	1	0.793	1	2.09E-05	2.68E-03	0.082	1
cg17944885	*ZNF788P, ZNF625-ZNF20*	0.756	1	0.034	1	3.72E-13	4.76E-11	0.894	1
cg05165263	*IRF2*	0.232	1	0.659	1	6.62E-05	8.47E-03	0.788	1
cg05710777	*LINC01800*	0.601	1	0.093	1	0.991	1	0.762	1
cg12864625	*MBNL1*	0.363	1	0.709	1	0.71	1	0.144	1
cg03026982	*NAV2*	0.228	1	0.667	1	0.39	1	0.558	1
cg10473623	*NAV2*	0.670	1	0.839	1	0.749	1	0.431	1
cg08150816	*NME7*	NA	NA	NA	NA	NA	NA	NA	NA
cg06587767	*PIP5K1C*	0.903	1	0.947	1	0.283	1	0.455	1
cg24382141	*PSKH1*	0.022	1	0.02	1	0.002	0.256	0.448	1
cg15167811	*PTBP3*	0.766	1	0.922	1	0.684	1	0.574	1
cg00008629	*PTBP3*	0.678	1	0.187	1	0.239	1	0.437	1
cg23527387	*REV1*	0.400	1	0.667	1	0.26	1	0.252	1
cg02711608	*SLC1A5*	NA	NA	NA	NA	NA	NA	NA	NA
cg21961721	*SLC27A3*	0.400	1	0.613	1	<2.20E-308	<2.20E-308	0.221	1
cg08230697	*STAB2*	0.115	1	0.808	1	0.208	1	0.245	1
cg02917536	*TAB2*	0.558	1	0.327	1	0.018	1	0.497	1
cg19693031	*TXNIP*	6.22E-14	7.96E-12	0.055	1	0.752	1	0.132	1
cg11414254	*ZNF346*	0.903	1	0.662	1	0.38	1	0.582	1

P_{adj} is the *p*-value adjusted for the number of lookups (32 CpGs × four phenotypes). CRIC=Chronic Renal Insufficiency Cohort, eGFR=estimated glomerular filtration rate. HbA_{1c}=glycated haemoglobin

Supplementary Table 4 eFORGE-TF results (p<x10^{-5}) for motif overlap with DKD-associated differentially methylated CpG sites.

CpG	Gene	TF id	TF name	Database	CHR	FIMO (p)
cg22815707	*ANKRD12*	HOMEZ_HOMEZ_1	Homeobox and leucine zipper protein Homez (HOMEZ)	Taipale/SELEX	chr18:9138599-9138611: -	6.30E-06
cg17058475	*CPT1A*	V_PPARG_02	Peroxisome proliferator-activated receptor gamma (PPARG)	TRANSFAC	chr11:68607722-68607745: +	9.52E-06
cg05165263	*IRF2*	V_PAX3_01	Paired box protein Pax-3 (PAX3)	TRANSFAC	chr4:185317216-185317229: -	5.42E-06
cg05710777	*LINC01800*	Tcfap2a_secondary	Transcription factor AP-2-alpha (TFAP2A)	UniProbe	chr2:65089812-65089826: -	3.58E-06
cg01895164	*Intergenic (PAFAH2, EXTL1)*	V_P53_04	Cellular tumor antigen p53 (P53)	TRANSFAC	chr1:26332143-26332163: +	1.08E-06
		V_CMYB_Q5	Transcriptional activator Myb (MYB)	TRANSFAC	chr1:26332144-26332155: -	5.33E-06
		V_P53_04	Cellular tumor antigen p53 (P53)	TRANSFAC	chr1:26332143-26332163: -	5.47E-06
		V_P53_01	Cellular tumor antigen p53 (P53)	TRANSFAC	chr1:26332143-26332163: +	7.71E-06
cg10072464	*Intergenic (GRHL1, KLF11)*	VDR_nuclearreceptor_1	Vitamin D3 receptor (VDR)	Taipale/SELEX	chr13:114064535-114064551: +	2.44E-07
		Vdr.mouse_nuclearreceptor_1	Vitamin D3 receptor (VDR)	Taipale/SELEX	chr13:114064535-114064551: +	4.89E-07
		V_DR3_Q4	Vitamin D3 receptor (VDR)	TRANSFAC	chr13:114064519-114064540: +	3.09E-06
cg19996939		V_SREBP_Q6	Sterol regulatory element-binding proteins (SREBPs)	TRANSFAC	chr6:135466478-135466493: -	2.92E-06
		V_SREBP1_Q5	Sterol regulatory element-binding proteins (SREBPs)	TRANSFAC	chr6:135466478-135466493: -	5.38E-06

Data from 850k array and fKidney samples, 7 experiments. Abbreviations: CHR=Chromosome, FIMO=Find Indvidual Motif Occurrences, TF=Transcription factor, TRANSFAC=TRANScription FACtor database

Supplementary Table 5 Differential kidney gene expression for DKD in the NephroSeq database v4

**Dataset name**	ERCB Nephrotic Syndrome TubInt	Ju CKD Glom	Ju CKD Glom	Ju CKD TubInt	Schmid Diabetes TubInt	Schmid Diabetes TubInt	Woroniecka Diabetes Glom	Woroniecka Diabetes TubInt
**Group:**	DKD vs. Healthy LD	DKD vs. Healthy LD	DKD vs. Other Diseases	DKD vs. Healthy LD	DKD vs. MCD	DKD vs. MCD and Control	DN vs. Healthy LD	DN vs. Healthy LD
ANKRD12							FC= -1.602 (p = 0.003)	
FKBP5	FC = -3,303 (p = 0.001)	FC = -3.233 ($p = 5.76\times10^{-10}$)	FC = -1.64 ($p = 6.55\times10^{-5}$)	FC = -3.272 ($p = 4.06\times10^{-8}$)	FC = -2.176 ($p = 1.21\times10^{-4}$)			FC = -1.534 ($p = 7.00\times10^{-4}$)
GRK5							FC = -4.343 ($p = 9.49\times10^{-7}$)	FC = 1.566 (p = 0.026)
INPP4B								FC = 1.514 (p = 0.012)
MBNL1								FC = 1.620 (p = 0.005)
NME7							FC = -2.485 ($p = 5.51\times10^{-4}$)	
PTBP3								FC = 2.587 ($p = 1.20\times10^{-5}$)
REV1							FC = -1.604 $p = 2.90\times10^{-4}$	
SLC27A3		FC = 1.565 ($p = 6.61\times10^{-5}$)						
TXNIP					FC = 1.665 (p = 0.009)	FC = 1.598 (p = 0.004)	FC = -1.503 ($p = 1.84\times10^{-4}$)	FC = 3.137 ($p = 4.67\times10^{-4}$)

Results searched for for genes containing DKD-associated CpGs. Only those population groups which assessed DKD are included. Any genes with no results have been removed from this table. Abbreviations: CKD=Chronic Kidney Disease, DKD=Diabetic kidney disease, DN=Diabetic nephropathy, ERCB=European Renal cDNA Bank, Glom=Glomeruli, LD=Living Donor, MCD=Minimal Change Disease, TubInt=Tubulointerstitium

Supplementary Table 6 Differential kidney gene expression in the North Dublin Renal Biobank RNA-Seq data

Gene	*p*-value	Adj *p*-value	Log_2 Ratio	Analysis	Comparison	Participants
C5orf66	0.002	0.028	0.362	DE	CRP, median value as threshold	*n*=39, native and transplant
GJA5	5.47E-05	0.011	-0.795	DE	eGFR, median value as threshold	*n*=39, native and transplant
GJA5	0.007	0.041	-0.68	DE	Fibrosis, median value as threshold	*n*=39, native and transplant
GJA5	5.48E-05	0.011	-0.795	DE	eGFR, median value as threshold	*n*=24, native kidney only
INPP4B	0.0003	0.037	-0.581	DE	eGFR, median value as threshold	*n*=44, native and transplant
INPP4B	0.008	0.043	0.483	DE	Fibrosis, median value as threshold	*n*=39, native and transplant
INPP4B	0.003	0.037	0.712	DE	Fibrosis, median value as threshold	*n*=24, native kidney only
IRF2	0.001	0.027	0.298	DE	Fibrosis, median value as threshold	*n*=24, native kidney only
MBNL1	0.003	0.022	0.452	DE	Fibrosis, median value as threshold	*n*=39, native and transplant
MBNL1	0.007	0.035	0.417	DE	Progression to kidney failure vs. no progression	*n*=44, native and transplant
MBNL1	0.0004	0.022	0.531	DE	Fibrosis, median value as threshold	*n*=24, native kidney only
NAV2	1.02E-06	0.001	-0.31	DE	CRP, median value as threshold	*n*=39, native and transplant
PSKH1	0.006	0.034	0.408	Correlation	Log eGFR at baseline	*n*=44, native and transplant
PTBP3	0.003	0.022	0.492	DE	Fibrosis, median value as threshold	*n*=39, native and transplant
PTBP3	0.003	0.026	-0.581	Correlation	Log eGFR at baseline	*n*=24, native kidney only
SLC1A5	0.0002	0.008	0.682	Correlation	Log eGFR at baseline	*n*=24, native kidney only
SLC27A3	0.001	0.015	-0.465	Correlation	Log eGFR at baseline	*n*=44, native and transplant
SLC27A3	0.002	0.036	0.476	DE	eGFR, median value as threshold	*n*=23, native kidney only
TXNIP	1.75E-05	0.004	0.806	DE	Fibrosis, median value as threshold	*n*=39, native and transplant
TXNIP	1.13E-05	0.002	0.974	DE	Progression to kidney failure vs. no progression	*n*=44, native and transplant
TXNIP	0.0002	0.008	-0.682	Correlation	Log eGFR at baseline	*n*=24, native kidney only
TXNIP	0.0009	0.025	0.717	DE	Fibrosis, median value as threshold	*n*=24, native kidney only

Abbreviations: Adj=adjusted (for age and sex), CRP=C reactive protein, DE=differential expression, eGFR=estimated glomerular filtration rate

Supplementary Table 7 Kidney gene expression correlations in American Indian T2D population with DKD (n=97)

Gene	Description	Correlation (Glomerular)	Correlation (Tubular)
ANKRD12	percentage of intact foot processes on both the peripheral and mesangial glomerular basement membrane	*r* = 0.274 (*p* = 0.026)	
ANKRD12	percent of endothelial fenestration falling on the peripheral glomerular basement membrane	*r* = 0.285 (*p* = 0.020)	
FKBP5	Albumin-creatinine ratio	*r* = -0.278 (*p* = 0.023)	
GJA5	glomerular basement membrane width	*r* = -0.279 (*p* = 0.023)	
GJA5	percent of endothelial fenestration falling on the peripheral glomerular basement membrane	*r* = 0.247 (*p* = 0.046)	
GJA5	surface volume of peripheral glomerular basement membrane per glomerulus	*r* = 0.282 (*p* = 0.022)	
GRK5	surface volume of peripheral glomerular basement membrane per glomerulus	*r* = -0.284 (*p* = 0.021)	
GRK5	glomerular filtration rate, slope		*r* = -0.451 (*p* = 0.001)
INPP4B	glomerular filtration rate, slope	*r* = -0.330 (*p* = 0.006)	
INPP4B	volume fraction of podocyte cell per glomerulus	*r* = -0.321 (*p* = 0.009)	
INPP4B	numerical density of podocyte cell per glomerulus	*r* = -0.239 (*p* = 0.053)	
INPP4B	Albumin-creatinine ratio		*r* = 0.353 (*p* = 0.015)
IRF2	glomerular filtration rate slope		*r* = -0.355 (*p* = 0.014)
MBNL1	numerical density of podocyte cell per glomerulus	*r* = -0.339 (*p* = 0.005)	
NAV2	glomerular basement membrane width	*r* = 0.289 (*p* = 0.019),	
NAV2	surface volume of peripheral glomerular basement membrane per glomerulus	*r* = -0.277 (*p* = 0.024)	
PIP5K1C	percent of endothelial fenestration falling on the peripheral glomerular basement membrane	*r* = -0.240 (*p* = 0.052),	
PIP5K1C	mesangial fractional volume	*r* = 0.290 (*p* = 0.018)	
PTBP3	foot process width in peripheral glomerular basement membrane	*r* = -0.309 (p = 0.011)	
REV1	glomerular filtration rate Slope	*r* = 0.326 (*p* = 0.007)	
REV1	Haemoglobin A1c		*r* = -0.296 (*p* = 0.043)
SLC1A5	percent of endothelial fenestration falling on the peripheral glomerular basement membrane	*r* = -0.296 (*p* = 0.016)	
SLC1A5	Age		*r* = -0.328 (*p* = 0.025)
SLC27A3	glomerular filtration rate, slope	*r* = -0.308 (*p* = 0.011)	
SLC27A3	numerical density of podocyte cell per glomerulus	*r* = -0.294 (*p* = 0.017)	
SLC27A3	Albumin-creatinine ratio		*r* = 0.293 (*p* = 0.046)
STAB2	glomerular filtration rate		*r* = -0.394 (*p* = 0.006)
TAB2	percent of endothelial fenestration falling on the peripheral glomerular basement membrane	*r* = 0.265 (*p* = 0.031)	
TAB2	glomerular filtration rate		*r* = 0.288 (*p* = 0.049)
TXNIP	percentage of intact foot processes on both the peripheral and mesangial glomerular basement membrane	*r* = -0.284 (*p* = 0.021),	
TXNIP	percent of endothelial fenestration falling on the peripheral glomerular basement membrane	*r* = 0.243 (*p* = 0.049)	

Glomerular refers to glomerular kidney tissue and tubular to tubular kidney tissue. Abbreviations: DKD= Diabetic kidney disease, T2D=type 2 diabetes. Correlations are spearman correlations.

Supplementary Table 8 Mendelian randomisation results for the association between methylation at DKD-associated CpGs and DKD

Gene	Outcome	Exposure	Method	SNPs (*n*)	OR (95%CI)	*p*	Heterogeneity *p*	Egger intercept	Intercept *p*
ADPRHL1	DKD	cg10072464	IVW	4	1.10 (0.84-1.43)	0.506	0.07	0.06	0.56
FKBP5	DKD	cg03546163	IVW	2	0.79 (0.50-1.26)	0.329			
INPP4B	DKD	cg18376497	IVW	3	1.10 (0.88-1.38)	0.383	0.45	-0.08	0.45
PTBP3	DKD	cg00008629	Wald ratio	1	1.04 (0.61-1.78)	0.891			
REV1	DKD	cg23527387	Wald ratio	1	0.74 (0.58-0.94)	0.012			
TXNIP	DKD	cg19693031	Wald ratio	1	1.04 (0.89-1.22)	0.623			
ZNF20	DKD	cg17944885	IVW	4	1.00 (0.87-1.14)	0.997	0.71	0.02	0.67

IVW=inverse variance weighted regression, DKD =Diabetic kidney disease

Supplementary Table 9 Differentially methylated genes derived from METAL analysis from three models

Gene	Location	Adjustment Model	*n*	Zscore	*p*	Direction	p_{het}	Sites (*n*)
ENSG00000235575 (RP4-800F24.1)	1:169279903-169291717	Minimal Model	1290	-6.096	1.09E-09	--	0.18	2
ENSG00000235575 (RP4-800F24.1)	1:169279903-169291717	Minimal + Current Smoking Status Model	1166	-5.681	1.338E-08	--	0.16	2
ENSG00000268863 (AC135048.1)	16:30996808-30997533	Minimal Model	1291	-4.978	6.44E-07	--	0.02	1
ENSG00000268863 (AC135048.1)	16:30996808-30997533	Minimal + Current Smoking Status Model	1166	-4.702	2.57E-06	--	0.03	1
ENSG00000268863 (AC135048.1)	16:30996808-30997533	Maximal Model	952	-4.906	9.29E-07	--	0.11	1

Results with $p \leq x10^{-5}$ reported. The significance threshold for epigenome-wide significance: $p \leq 1x10^{-8}$.

Supplementary Table 10 Genetics of Nephropathy – an International Effort (GENIE) members

Institute	Name	Affiliations
**Broad Institute, Cambridge, MA USA.<br>Massachusetts General Hospital, Boston, MA USA.<br>Harvard Medical School, Boston, MA USA<br>Boston Children's Hospital, Boston, MA, USA**		
	Jose C Florez	Programs in Metabolism and Medical & Population Genetics, Broad Institute, Cambridge, MA USA<br>Diabetes Unit and Center for Genomic Medicine, Massachusetts General Hospital, Boston, MA USA.<br>Department of Medicine, Harvard Medical School, Boston, MA USA.
	Joel N Hirschhorn	Programs in Metabolism and Medical & Population Genetics, Broad Institute, Cambridge, MA USA.<br>Division of Endocrinology, Boston Children's Hospital, Boston, MA, USA<br>Department of Pediatrics and Genetics, Harvard Medical School, Boston, MA, USA
	Joanne B Cole	Department of Biomedical Informatics, University of Colorado School of Medicine.<br>Programs in Metabolism and Medical & Population Genetics, Broad Institute, Cambridge, MA USA.<br>Diabetes Unit and Center for Genomic Medicine, Massachusetts General Hospital, Boston, MA USA.
	Raymond Kreienkamp	Division of Endocrinology, Boston Children's Hospital, Boston, MA, USA<br>Diabetes Unit and Center for Genomic Medicine, Massachusetts General Hospital, Boston, MA USA.
**University Belfast, Belfast, UK**		
	Laura J Smyth	Molecular Epidemiology Research Group, Centre for Public Health, Queen's University Belfast, Belfast, UK.
	Katie Kerr	Molecular Epidemiology Research Group, Centre for Public Health, Queen's University Belfast, Belfast, UK.
	Jill Kilner	Molecular Epidemiology Research Group, Centre for Public Health, Queen's University Belfast, Belfast, UK.
	Yogesh Gupta	Molecular Epidemiology Research Group, Centre for Public Health, Queen's University Belfast, Belfast, UK.
	Claire Hill	Molecular Epidemiology Research Group, Centre for Public Health, Queen's University Belfast, Belfast, UK.
	Christopher Wooster	Molecular Epidemiology Research Group, Centre for Public Health, Queen's University Belfast, Belfast, UK.
	Kerry Anderson	Molecular Epidemiology Research Group, Centre for Public Health, Queen's University Belfast, Belfast, UK.
	Gareth J McKay	Molecular Epidemiology Research Group, Centre for Public Health, Queen's University Belfast, Belfast, UK.
	Amy Jayne McKnight	Molecular Epidemiology Research Group, Centre for Public Health, Queen's University Belfast, Belfast, UK.
	Alexander P Maxwell	Molecular Epidemiology Research Group, Centre for Public Health, Queen's University Belfast, Belfast, UK.<br>Regional Nephrology Unit, Belfast City Hospital, Belfast, Northern Ireland, UK.
**The FinnDiane Study Group<br>Folkhälsan Research Center, Helsinki, Finland.<br>University of Helsinki and Helsinki University Hospital, Helsinki, Finland.<br>University of Helsinki, Helsinki, Finland.**		
	Emma H Dahlström	Folkhälsan Institute of Genetics, Folkhälsan Research Center, Helsinki, Finland.<br>Department of Nephrology, University of Helsinki and Helsinki University Hospital, Helsinki, Finland.<br>Research Program for Clinical and Molecular Metabolism, Faculty of Medicine, University of Helsinki, 00290, Helsinki, Finland.
	Anna Syreeni	Folkhälsan Institute of Genetics, Folkhälsan Research Center, Helsinki, Finland.<br>Department of Nephrology, University of Helsinki and Helsinki University Hospital, Helsinki, Finland.

		Research Program for Clinical and Molecular Metabolism, Faculty of Medicine, University of Helsinki, 00290, Helsinki, Finland.
	Erkka Valo	Folkhälsan Institute of Genetics, Folkhälsan Research Center, Helsinki, Finland.<br>Department of Nephrology, University of Helsinki and Helsinki University Hospital, Helsinki, Finland.<br>Research Program for Clinical and Molecular Metabolism, Faculty of Medicine, University of Helsinki, 00290, Helsinki, Finland.
	Carol Forsblom	Folkhälsan Institute of Genetics, Folkhälsan Research Center, Helsinki, Finland.<br>Department of Nephrology, University of Helsinki and Helsinki University Hospital, Helsinki, Finland.<br>Research Program for Clinical and Molecular Metabolism, Faculty of Medicine, University of Helsinki, 00290, Helsinki, Finland.<br>Deceased.
	Niina Sandholm	Folkhälsan Institute of Genetics, Folkhälsan Research Center, Helsinki, Finland.<br>Department of Nephrology, University of Helsinki and Helsinki University Hospital, Helsinki, Finland.<br>Research Program for Clinical and Molecular Metabolism, Faculty of Medicine, University of Helsinki, 00290, Helsinki, Finland.
	Per-Henrik Groop	Folkhälsan Institute of Genetics, Folkhälsan Research Center, Helsinki, Finland.<br>Department of Nephrology, University of Helsinki and Helsinki University Hospital, Helsinki, Finland.<br>Research Program for Clinical and Molecular Metabolism, Faculty of Medicine, University of Helsinki, 00290, Helsinki, Finland.<br>Department of Diabetes, Central Clinical School, Monash University, Melbourne, Victoria, Australia.
**University College Dublin, Dublin Ireland**		
	Ross Doyle	Diabetes Complications Research Centre, Conway Institute, School of Medicine, University College Dublin, Dublin Ireland.
	Eoin Brennan	Diabetes Complications Research Centre, Conway Institute, School of Medicine, University College Dublin, Dublin Ireland.
	Darrell Andrews	Diabetes Complications Research Centre, Conway Institute, School of Medicine, University College Dublin, Dublin Ireland.
	Denise Sadlier	Mater Misericordiae Hospital, Dublin, Ireland D07 K201.
	Finian Martin	Diabetes Complications Research Centre, Conway Institute, School of Medicine, University College Dublin, Dublin Ireland.
	Catherine Godson	Diabetes Complications Research Centre, Conway Institute, School of Medicine, University College Dublin, Dublin Ireland.
**University of Michigan School of Medicine, Ann Arbor, MI, USA**		
	Viji Nair	Department of Medicine-Nephrology, University of Michigan School of Medicine, Ann Arbor, MI 48109, USA.
	Damian Fermin	Department of Pediatrics-Nephrology, University of Michigan School of Medicine, Ann Arbor, MI 48109, USA.
	Lalita Subramanian	Department of Medicine-Nephrology, University of Michigan School of Medicine, Ann Arbor, MI 48109, USA.
	Matthias Kretzler	Department of Internal Medicine, University of Michigan, Ann Arbor, Michigan, USA.
**University of Pennsylvania, Perelman School of Medicine, Philadelphia, PA, USA.**		
	Hongbo Liu	Renal, Electrolyte, and Hypertension Division, Department of Medicine, University of Pennsylvania, Perelman School of Medicine, Philadelphia, PA, USA.<br>Institute for Diabetes, Obesity, and Metabolism, University of Pennsylvania, Perelman School of Medicine, Philadelphia, PA, USA.<br>Department of Genetics, University of Pennsylvania, Perelman School of Medicine, Philadelphia, PA, USA
	Katalin Susztak	Renal, Electrolyte, and Hypertension Division, Department of Medicine, University of Pennsylvania, Perelman School of Medicine, Philadelphia, PA, USA.<br>Institute for Diabetes, Obesity, and Metabolism, University of Pennsylvania, Perelman School of Medicine, Philadelphia, PA, USA.

		Department of Genetics, University of Pennsylvania, Perelman School of Medicine, Philadelphia, PA, USA
**University of California San Diego, La Jolla, CA, USA**		
	Rany M Salem	Herbert Wertheim School of Public Health and Human Longevity Science, University of California San Diego, La Jolla, CA, USA

Supplementary Figures

Supplementary Figure 1 Representative concordance plots for a duplicate sample pair.
Average **r2 for seven duplicates = 0.99.**

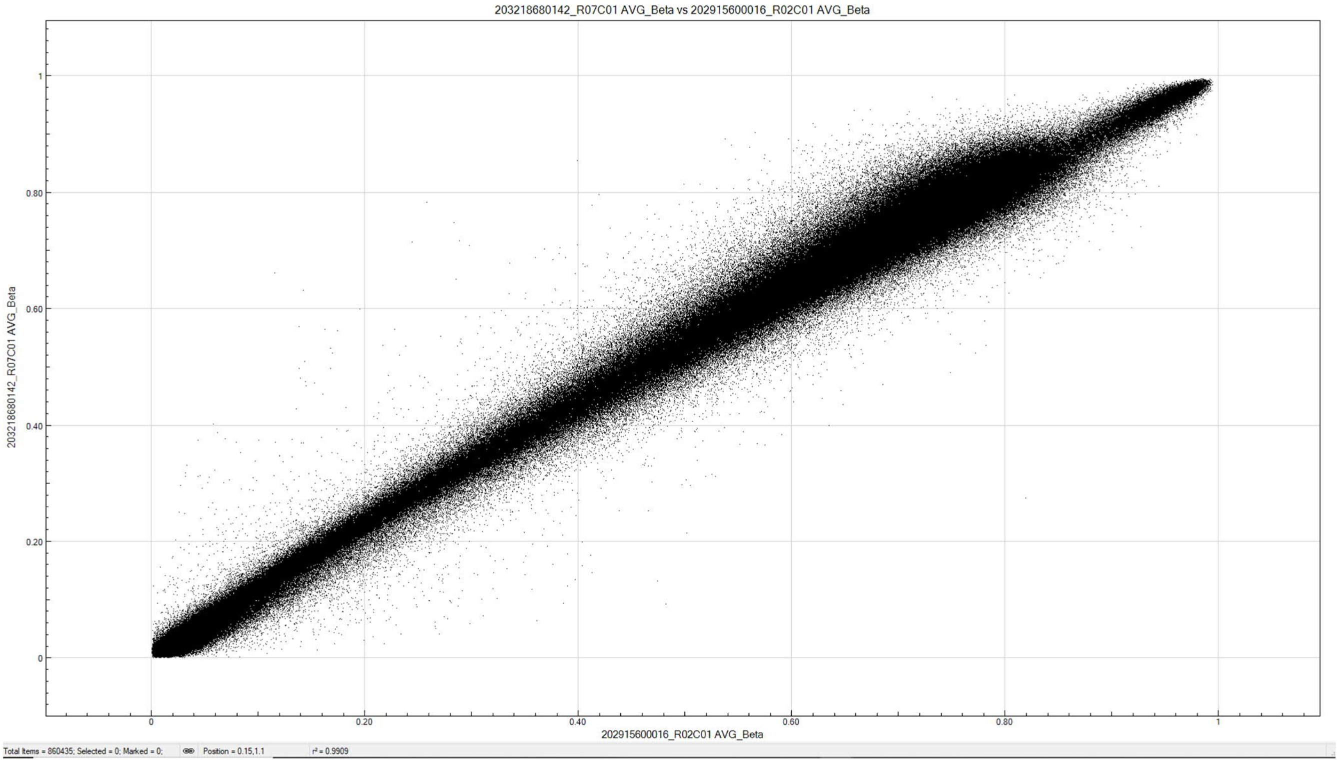

Supplementary Figure 2 Flow chart depicting the methods undertaken

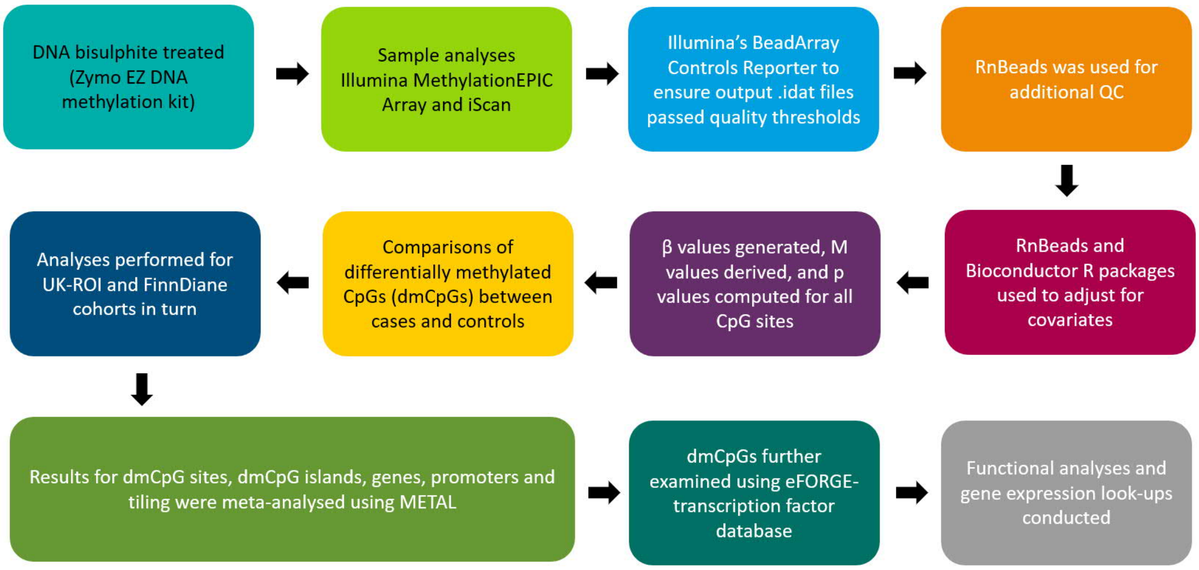

Abbreviations: DKD=Diabetic kidney disease, FinnDiane=Finnish Diabetic Nephropathy study, QC=Quality control, ROI=Republic of Ireland, UK=United Kingdom.

Supplementary Figure 3 Regional association plots (A-G) for the seven CpGs EWAS-significant in each meta-analysis model.
The regional plots included cg21961721 within *SLC27A3* (A), cg08150816 within *NME7* (B), cg05710777 within *LINC01800* (C), cg00008629 and cg15167811within *PTBP3* (D) cg02711608 within *SLC1A5* (E) cg17944885 and

cg25544931 within a zinc finger gene region on chromosome 9 (F). Regional association plots were created using R package ‘coMet’ with gene track from Ensembl and CpG island and GWAS catalogue single nucleotide polymorphisms (SNPs) track from the UCSC. SNPs in red text have previously been associated with diabetic kidney disease. Circles represent CpGs and are coloured by their correlation (Spearman, r_s) to the lead CpG in the region (black circle) as follows: white: no correlation (p>0.05), shades of red: positive correlations, shades of blue: negative correlations. Correlations calculated based on methylation levels in the FinnDiane dataset (n=798). Association p-values were from the meta-analysis (minimal model adjusted for age, sex and six white blood cell counts), which combined FDR-adjusted p-values from the individual EWASs (in FinnDiane and UK-ROI). The p-values in the EWAS were computed from hierarchal linear models. EWAS significance was defined as $p<9.9\times10^{-8}$.

A.

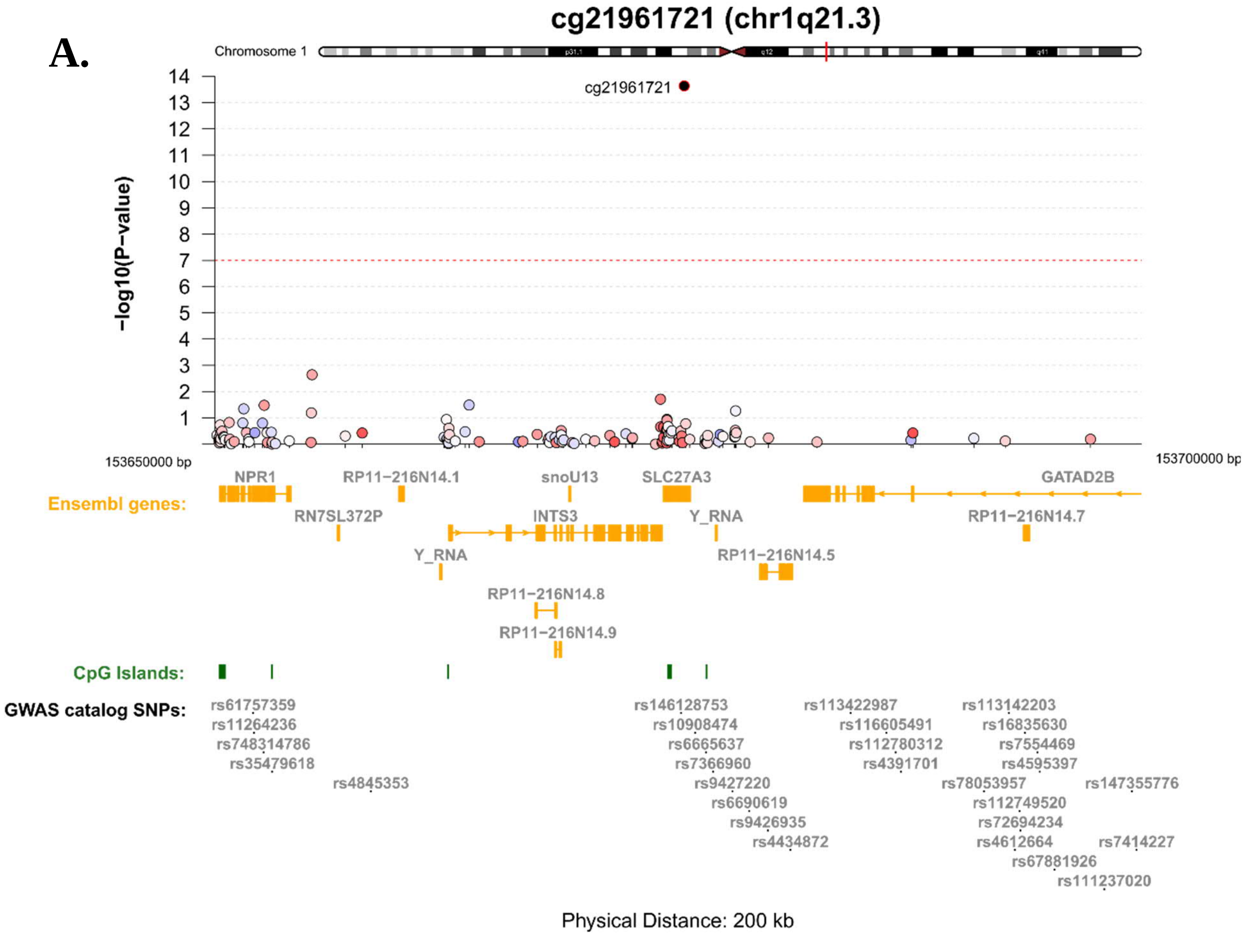

B.

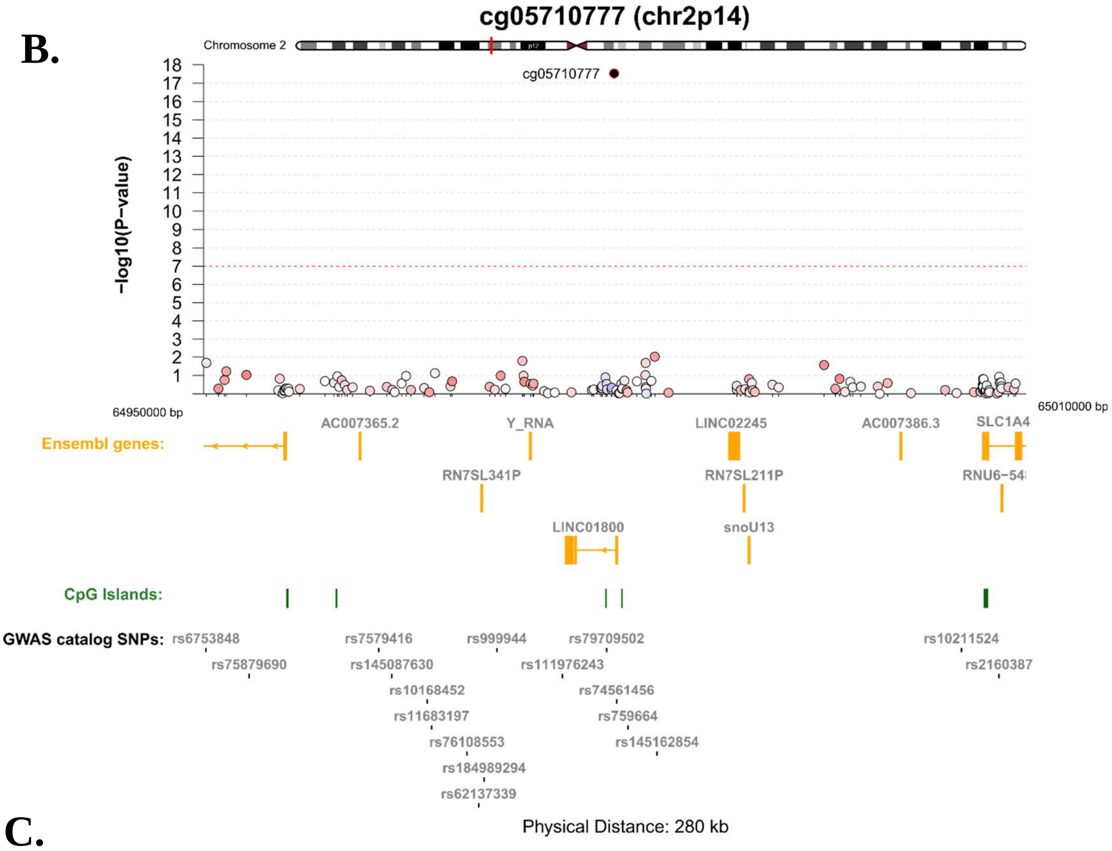
cg05710777 (chr2p14)
Chromosome 2
−log10(P−value)
cg05710777
64950000 bp
65010000 bp
Ensembl genes:
AC007365.2
Y_RNA
LINC02245
AC007386.3
RN7SL341P
RN7SL211P
LINC01800
snoU13
CpG Islands:
GWAS catalog SNPs:
rs6753848
rs75879690
rs7579416
rs145087630
rs10168452
rs11683197
rs76108553
rs184989294
rs62137339
rs999944
rs79709502
rs111976243
rs74561456
rs759664
rs145162854
rs10211524
rs2160387
Physical Distance: 280 kb

C.

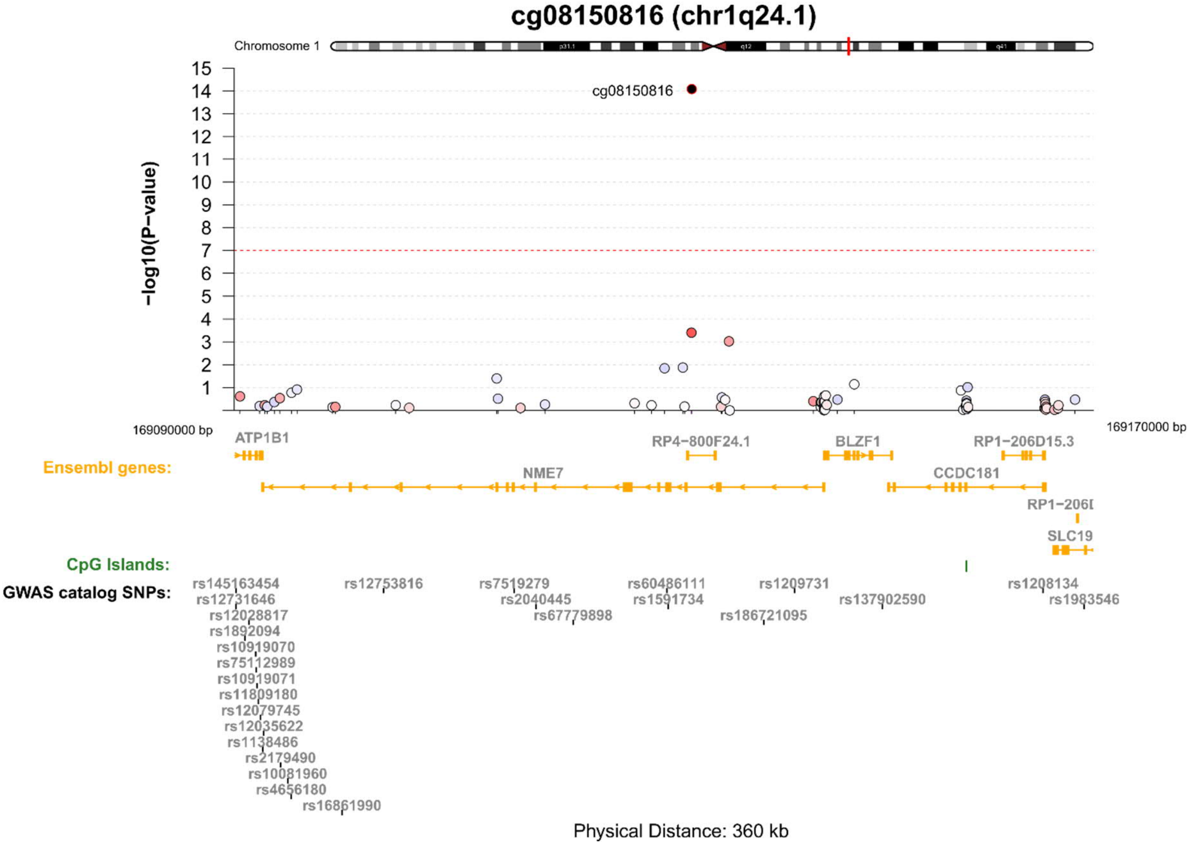
cg08150816 (chr1q24.1)
Chromosome 1
−log10(P−value)
cg08150816
169090000 bp
169170000 bp
Ensembl genes:
ATP1B1
RP4−800F24.1
BLZF1
RP1−206D15.3
NME7
CCDC181
CpG Islands:
GWAS catalog SNPs:
rs145163454
rs12731646
rs12028817
rs1892094
rs10919070
rs75112989
rs10919071
rs11809180
rs12079745
rs12035622
rs1138486
rs2179490
rs10081960
rs4656180
rs16861990
rs12753816
rs7519279
rs2040445
rs67779898
rs60486111
rs1591734
rs1209731
rs186721095
rs137902590
rs1208134
rs1983546
Physical Distance: 360 kb

D.

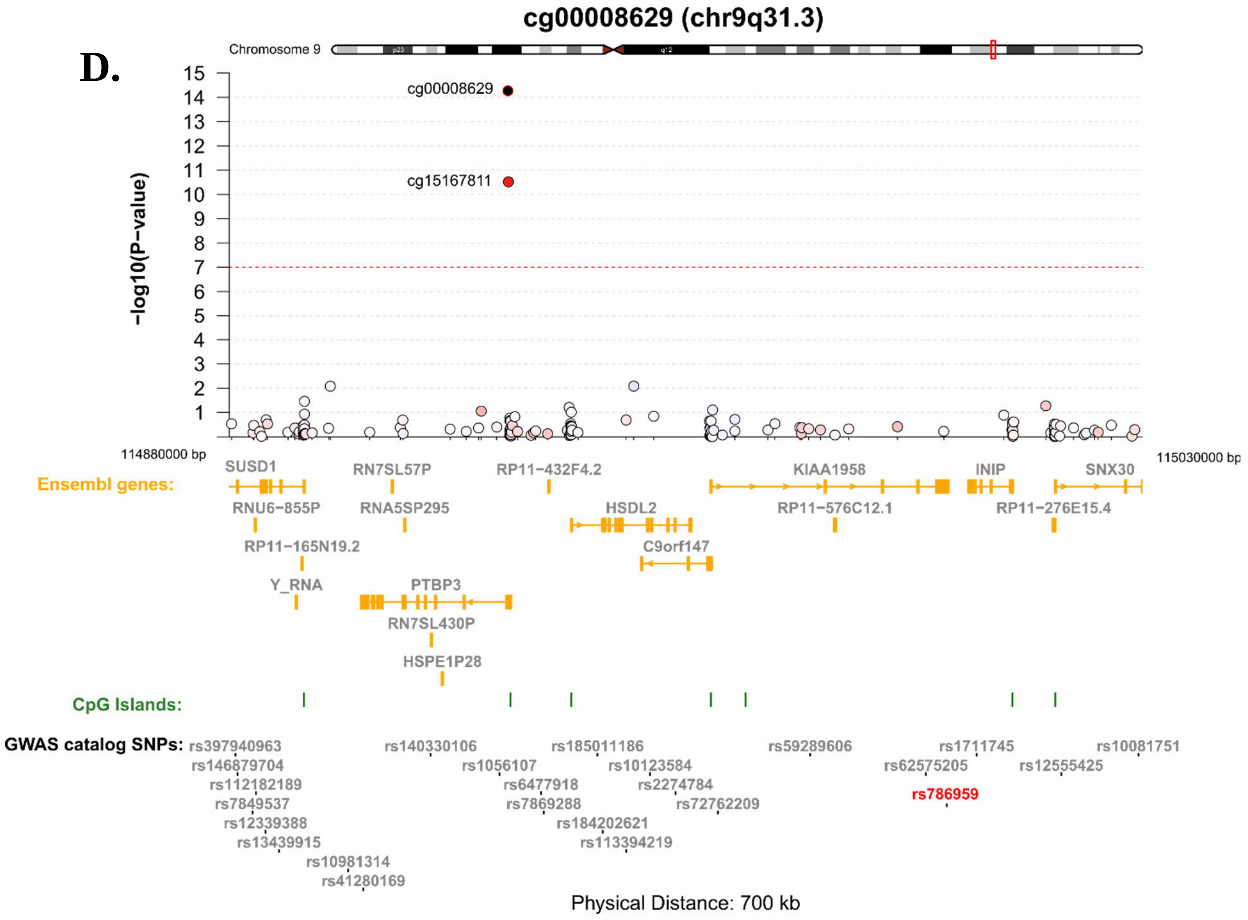
cg00008629 (chr9q31.3)
Chromosome 9
cg00008629
cg15167811
-log10(P-value)
114880000 bp
115030000 bp
Ensembl genes:
SUSD1
RN7SL57P
RP11-432F4.2
KIAA1958
INIP
SNX30
RNU6-855P
RNA5SP295
HSDL2
RP11-576C12.1
RP11-276E15.4
RP11-165N19.2
C9orf147
Y_RNA
PTBP3
RN7SL430P
HSPE1P28
CpG Islands:
GWAS catalog SNPs:
rs397940963
rs146879704
rs112182189
rs7849537
rs12339388
rs13439915
rs10981314
rs41280169
rs140330106
rs1056107
rs6477918
rs7869288
rs185011186
rs10123584
rs2274784
rs184202621
rs113394219
rs72762209
rs59289606
rs1711745
rs62575205
rs786959
rs10081751
rs12555425
Physical Distance: 700 kb

E.

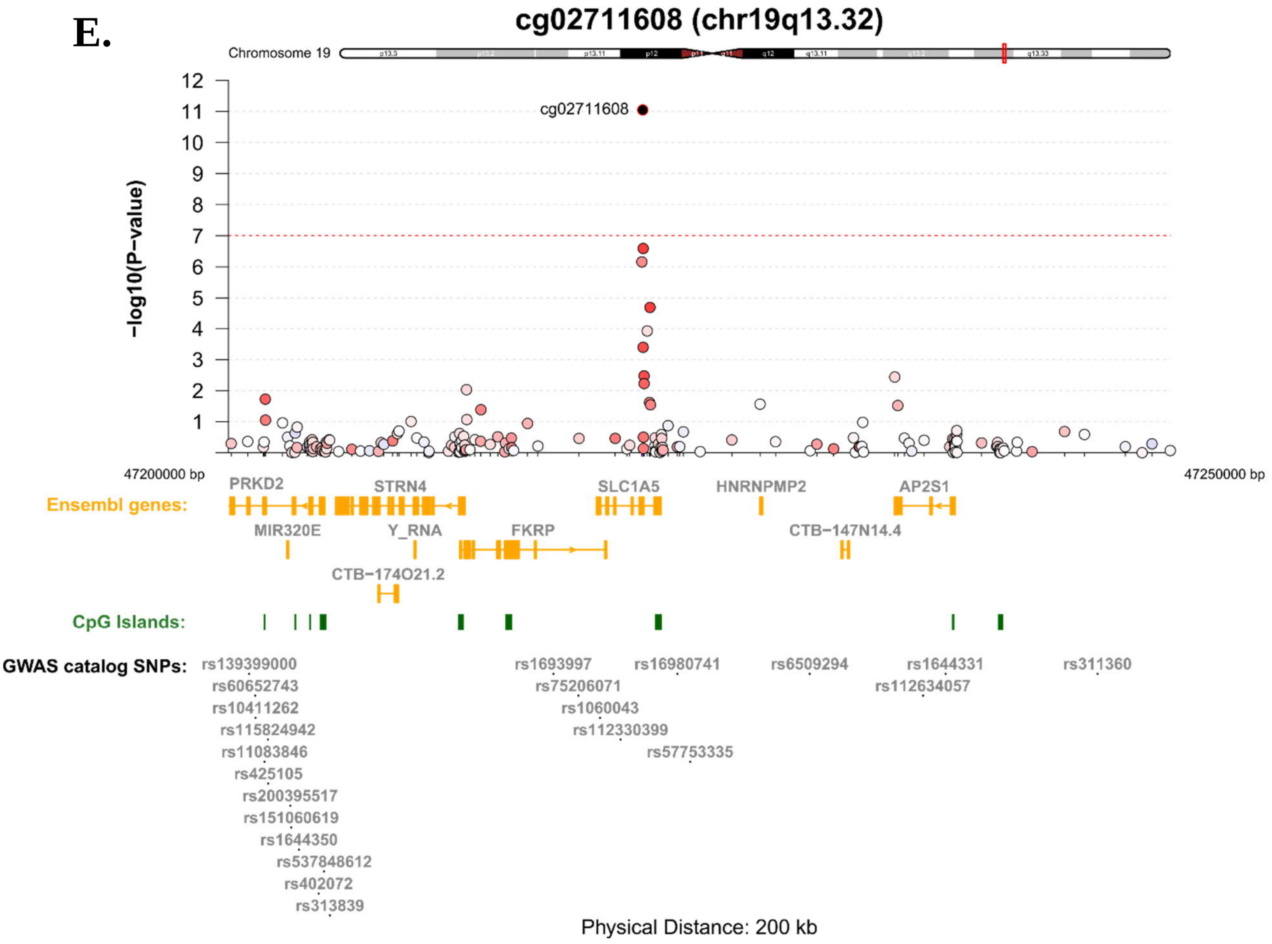
cg02711608 (chr19q13.32)
Chromosome 19
cg02711608
-log10(P-value)
47200000 bp
47250000 bp
Ensembl genes:
PRKD2
STRN4
SLC1A5
HNRNPMP2
AP2S1
MIR320E
Y_RNA
FKRP
CTB-147N14.4
CTB-174O21.2
CpG Islands:
GWAS catalog SNPs:
rs139399000
rs60652743
rs10411262
rs115824942
rs11083846
rs425105
rs200395517
rs151060619
rs1644350
rs537848612
rs402072
rs313839
rs1693997
rs75206071
rs1060043
rs112330399
rs57753335
rs16980741
rs6509294
rs1644331
rs112634057
rs311360
Physical Distance: 200 kb

F.

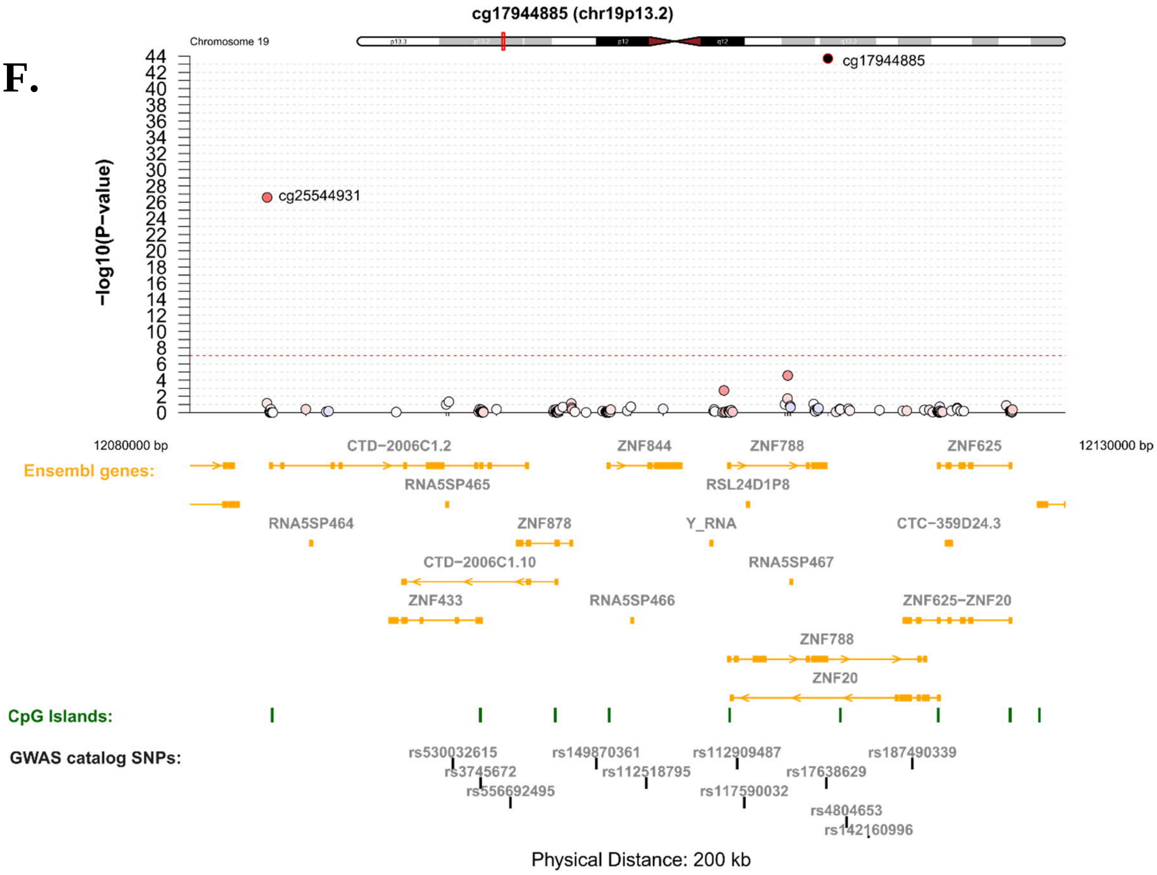
cg17944885 (chr19p13.2)
Chromosome 19
cg17944885
cg25544931
-log10(P-value)
12080000 bp
12130000 bp
Ensembl genes:
CTD-2006C1.2
ZNF844
ZNF788
ZNF625
RNA5SP465
RSL24D1P8
RNA5SP464
ZNF878
Y_RNA
CTC-359D24.3
CTD-2006C1.10
RNA5SP467
ZNF433
RNA5SP466
ZNF625-ZNF20
ZNF788
ZNF20
CpG Islands:
GWAS catalog SNPs:
rs530032615
rs149870361
rs112909487
rs187490339
rs3745672
rs112518795
rs17638629
rs556692495
rs117590032
rs4804653
rs142160996
Physical Distance: 200 kb

Supplementary Figure 4 Regional association plots for the DKD-associated CpG located within the ***REV1* gene**
Regional association plot was created using R package ‘coMet’ with gene track from ensembl and CpG island and GWAS catalogue single nucleotide polymorphisms (SNPs) track from the UCSC. SNPs in red text have previously been associated with diabetic kidney disease. Circles represent CpGs and are coloured by their correlation (Spearman, r_s) to the lead CpG in the region (black circle) as follows: white: no correlation (p>0.05), shades of red: positive correlations, shades of blue: negative correlations. Correlations calculated based on methylation levels in the FinnDiane dataset (n=798). Association p-values were from the meta-analysis (minimal model adjusted for age, sex and six white blood cell counts), which combined FDR-adjusted p-values from the individual EWASs (in FinnDiane and UK-ROI). The p-values in the EWAS were computed from hierarchal linear models.

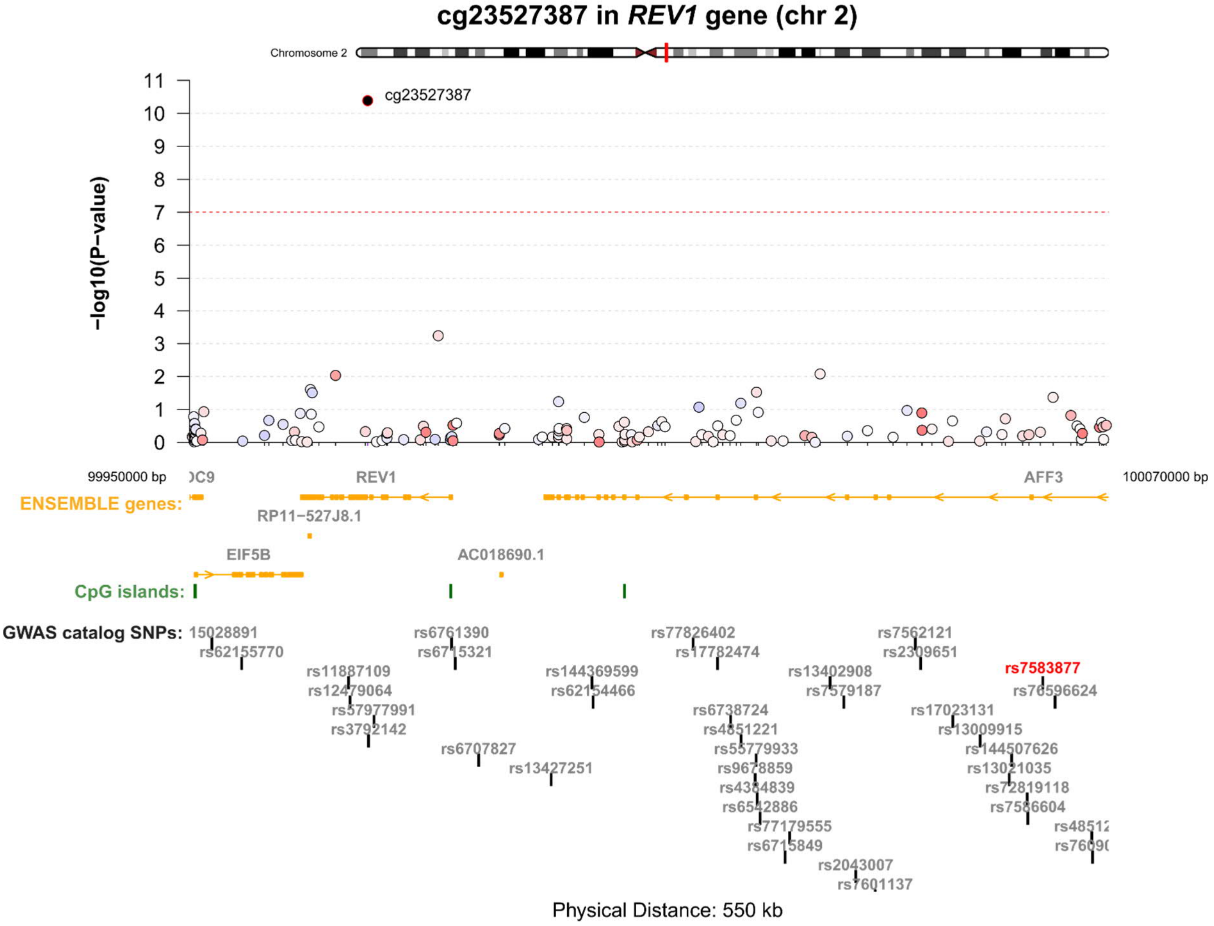

Supplement: Supplementary file 1 — Supplementary information [file 41467_2022_34963_MOESM1_ESM.pdf]
