# Supplementary material for: Epigenome-wide meta-analysis identifies DNA methylation biomarkers associated with diabetic kidney disease
Source: Nat Commun. 2022 Dec 22;13:7891. doi: 10.1038/s41467-022-34963-6 (PMC9780337; doi:10.1038/s41467-022-34963-6)
Supplement: Supplementary file 3 — Description of Additional Supplementary Files [file 41467_2022_34963_MOESM3_ESM.pdf]

Description of Additional Supplementary Files

File Name: Supplementary Data 1
Description: Results for differentially methylated CpG sites reaching epigenome-wide significance in one of the three models

File Name: Supplementary Data 2
Description: Prospective analysis of DKD cases in the FinnDiane: CpGs methylation as a risk factor for the development of kidney failure

File Name: Supplementary Data 3
Description: Cis-expression quantitative methylation site(cis-eQTM) lookup for the 32 DKD-associated CpGs

File Name: Supplementary Data 4
Description: Lookups of previously published CpGs associated with DKD and related outcomes in diabetes

File Name: Supplementary Data 5
Description: Differentially methylated tilings (p≤x10-5) from the thre METAL analysis models

File Name: Supplementary Data 6
Description: Gene information for genes containing DKD-associated CpGs
