# Supplementary material for: Epigenome-wide meta-analysis identifies DNA methylation biomarkers associated with diabetic kidney disease
Source: Nat Commun. 2022 Dec 22;13:7891. doi: 10.1038/s41467-022-34963-6 (PMC9780337; doi:10.1038/s41467-022-34963-6)
Supplement: Supplementary file 5 — Reporting Summary [file 41467_2022_34963_MOESM5_ESM.pdf]

nature portfolio

Corresponding author(s): Prof Amy Jayne McKnight, Ds Niina Sandholm

Last updated by author(s): Oct 28, 2022

Reporting Summary

Nature Portfolio wishes to improve the reproducibility of the work that we publish. This form provides structure for consistency and transparency in reporting. For further information on Nature Portfolio policies, see our Editorial Policies and the Editorial Policy Checklist.

Statistics

For all statistical analyses, confirm that the following items are present in the figure legend, table legend, main text, or Methods section.

n/a	Confirmed	
☐	☒	The exact sample size (*n*) for each experimental group/condition, given as a discrete number and unit of measurement
☐	☒	A statement on whether measurements were taken from distinct samples or whether the same sample was measured repeatedly
☐	☒	The statistical test(s) used AND whether they are one- or two-sided<br>*Only common tests should be described solely by name; describe more complex techniques in the Methods section.*
☐	☒	A description of all covariates tested
☐	☒	A description of any assumptions or corrections, such as tests of normality and adjustment for multiple comparisons
☐	☒	A full description of the statistical parameters including central tendency (e.g. means) or other basic estimates (e.g. regression coefficient) AND variation (e.g. standard deviation) or associated estimates of uncertainty (e.g. confidence intervals)
☐	☒	For null hypothesis testing, the test statistic (e.g. *F*, *t*, *r*) with confidence intervals, effect sizes, degrees of freedom and *P* value noted<br>*Give P values as exact values whenever suitable.*
☐	☒	For Bayesian analysis, information on the choice of priors and Markov chain Monte Carlo settings
☐	☒	For hierarchical and complex designs, identification of the appropriate level for tests and full reporting of outcomes
☐	☒	Estimates of effect sizes (e.g. Cohen's *d*, Pearson's *r*), indicating how they were calculated

Our web collection on statistics for biologists contains articles on many of the points above.

Software and code

Policy information about availability of computer code

Data collection	We used the Illumina Infinium MethylationEPIC BeadChip array to examine CpG sites. Methylation arrays were scanned using a dedicated iScan machine and initial QC was done with Illumina's BACR Software. Further QC was done with Illumina's GenomeStudio v1.8. Houseman estimates (Houseman, E. A. et a, 2012) l were calculated for the proportional white cell counts (WCCs) for each sample with minfi Bioconductor (v3.10) package
Data analysis	P-values for differentially methylathed CpGs between DKD cases and controls were computed using RnBeads (v. 2.6.0, R version 4.0) with the Linear Models for Microarray Data (Limma) method, which is adapted for use with methylation data. Cox proportional hazard models were used for calculating the predictive effect of methylation on DKD risk in R (v.4.1.3.) using R package survival (v3.2-13) as well as R package survcomp (v.1.44.1) to calculate concordance statistics. Genetic ontology (GO) and KEGG enrichment analyses were performed using the gometh function in the R (v4.0) using R package missmethyl (v1.28.0). Gene expression analyses perfomed in the Northern Dublin Renal Biobank were completed using cor.test function (correlations) in base R (v4.0.3) or R package 'Limma' (v3.46.0) was used for differential expression. Any custom code has been uploaded to https://github.com/EmmDah/EWAS-Meta-analysis-on-DKD-in-T1D. Mendelian randomization analyses were conducted in R (v 4.1) using R package TwoSampleMR (v 0.5.6).

For manuscripts utilizing custom algorithms or software that are central to the research but not yet described in published literature, software must be made available to editors and reviewers. We strongly encourage code deposition in a community repository (e.g. GitHub). See the Nature Portfolio guidelines for submitting code & software for further information.

Data

Policy information about availability of data

All manuscripts must include a data availability statement. This statement should provide the following information, where applicable:

- Accession codes, unique identifiers, or web links for publicly available datasets
- A description of any restrictions on data availability
- For clinical datasets or third party data, please ensure that the statement adheres to our policy

The summary data generated in this study (all three models) have been deposited at GENIE Updates | RenGenPECT (qub.ac.uk). Individual-level data for the study participants (participant-level genome-wide CpG methylation and clinical data for participants in UK-ROI and FinnDiane and gene expression data in American Indians with T2D) are not publicly available due to ethical and legal reasons and due to the consent provided by the participant at the time of data collection. Access, which is subject to local regulations, can be obtained upon reasonable request by contacting the following persons (FinnDiane study: niina.sandholm@helsinki.fi, UK-ROI: a.j.mcknight@qub.ac.uk, American Indian population of Pima Indians with T2D; vijin@med.umich.edu). Upon approval, analyses need to be performed on a local server (with protected, user-specific access) and requires signing non-disclosure and privacy agreements. The summary data used for the two-sample Mendelian randomization analyses are available on the GoDMC site (SNP-CpG associations; http://mqtldb.godmc.org.uk/) and on the type 2 diabetes knowledge portal (SNP-DKD associations; https://t2d.hugeamp.org/dinspector.html?dataset=GWAS_DNCRI). The data from the eforge TF database can be accessed on https://eforge-tf.altiusinstitute.org/. The nephroseq v4, containing the seven DKD kidney gene expression datasets (four cohorts), is a free platform to the academic and non-profit community and data deposited there can be analysed and accessed after registration and login (www.nephroseq.org). The nephroseq datasets 'Woroniecka Diabetes TubInt' (n=2) are also available under accession code http://www.ncbi.nlm.nih.gov/geo/query/acc.cgi?acc=GSE30122. The Northern Dublin Renal Biobank RNAseq data are available under accession code https://www.ncbi.nlm.nih.gov/geo/query/acc.cgi?acc=GSE137570. For the SNP-DKD associations the dataset 'late diabetic kidney disease (diabetic nephropathy)' was used. The eQTM data searched in this study is available on http://www.susztaklab.com/Kidney_meQTL/eQTM.php (kidney eQTM), https://static-content.springer.com/esm/art%3A10.1186%2Fs13148-021-01041-5/MediaObjects/13148_2021_1041_MOESM2_ESM.xlsx (whole blood eQTM, Framigham study) , http://bbmri.researchlumc.nl/atlas/#query (whole blood eQTM, BIOS consortium) and https://static-content.springer.com/esm/art%3A10.1186%2Fs12864-018-4842-3/MediaObjects/12864_2018_4842_MOESM2_ESM.txt (monocytes, eQTM).

Human research participants

Policy information about studies involving human research participants and Sex and Gender in Research.

Reporting on sex and gender	Biological sex was determined and checked with the Illumina GenomeStudio v1.8 from the methylation data. Cases and controls in both FinnDiane and UK-ROI were matched for sex. Sex was adjusted for in the analyses, and reported whenever possible.
Population characteristics	This study included 1,304 participants with T1DM (651 cases and 653 controls with and without DKD) from the United Kingdom - Republic of Ireland (UK-ROI, n=504) and the Finnish Diabetic Nephropathy (FinnDiane, n=800) study. 38% were men in FinnDiane, 52% in UK-ROI. Mean age was 43 in FinnDiane and 42 in UK-ROI. Individuals without DKD were matched with individuals with DKD based on age, sex, duration, and smoking.
Recruitment	FinnDiane participants are from the nationwide multicenter study, which is recruiting individuals with type 1 diabetes in 93 health care centers accross Finland. At a regular visit to the doctor, the individual is asked to participate in the FinnDiane study. This may, of course, create some bias of recruiting individuals who feel well enough to participate in further studies. The UK-ROI participants were Participants were recruited as part of the All Ireland-Warren 3-Genetics of Kidneys in Diabetes (GoKinD) United Kingdom (UK) Collection or the Belfast Renal Transplant Collection.
Ethics oversight	The ethical approval reference number for the UK-ROI GoKinD samples is MREC 175/23 (RO321) and for the Belfast Renal Transplant samples is ORECNI 08/NIR03/79, 12/NI/0003, 12/NI/0178. The FinnDiane study protocol was approved by the ethics committee of the Helsinki and Uusimaa Hospital District (HUS) (491/E5/2006, 238/13/03/00/2015, and HUS-3313-2018, July 3rd, 2019), and the study was performed in accordance with the Declaration of Helsinki. The North Dublin Renal Biobank study protocol was approved by Beaumont Hospital Ethics Committee. The study on American Indian population of Pima Indians with T2D was approved by the Institutional Review Board of the National Institute of Diabetes. and Digestive and Kidney Diseases. All participants gave their informed written consent prior participating.

Note that full information on the approval of the study protocol must also be provided in the manuscript.

Field-specific reporting

Please select the one below that is the best fit for your research. If you are not sure, read the appropriate sections before making your selection.

☒ Life sciences ☐ Behavioural & social sciences ☐ Ecological, evolutionary & environmental sciences

For a reference copy of the document with all sections, see nature.com/documents/nr-reporting-summary-flat.pdf

Life sciences study design

All studies must disclose on these points even when the disclosure is negative.

Sample size	We designed the study to include close to the maximum number of available DKD cases (400 in FinnDiane and XX in UKROI) and a matching number of controls with type 1 diabetes. We calculated that we had >80% power (pwrEWAS) to detect a true positive association from the EPIC array, defined as detecting CpGs with FDR-adjusted p-value ≤0.05
Data exclusions	We excluded participants without capacity to provide informed consent or if they did not provide informed consent for any reason and participants with non-White self-reported ethnicity
Replication	In lack of a replication cohort with methylome-wide data and information on diabetic kidney disease in type 1 diabetes, we used available published data from studies on diabetic kidney disease and any form of diabetes to verify our results. In addition, further functional data was used the verify the relevance of the identified CpGs (eQTM data in blood and kidney tissue) and gene expression data from external kidney tissue data sets (CKD and kidney disease in diabetes of unspecified type and type 2 diabetes.)
Randomization	The study design was observational and therefore randomization was not relevant to the study.
Blinding	The study design was observational and therefore blinding was not relevant to this study

Reporting for specific materials, systems and methods

We require information from authors about some types of materials, experimental systems and methods used in many studies. Here, indicate whether each material, system or method listed is relevant to your study. If you are not sure if a list item applies to your research, read the appropriate section before selecting a response.

Materials & experimental systems

n/a	Involved in the study
☒	☐ Antibodies
☒	☐ Eukaryotic cell lines
☒	☐ Palaeontology and archaeology
☒	☐ Animals and other organisms
☒	☐ Clinical data
☒	☐ Dual use research of concern

Methods

n/a	Involved in the study
☒	☐ ChIP-seq
☒	☐ Flow cytometry
☒	☐ MRI-based neuroimaging
